# Spammer detection using multi-classifier information fusion based on evidential reasoning rule

**DOI:** 10.1038/s41598-022-16576-7

**Published:** 2022-07-21

**Authors:** Shuaitong Liu, Xiaojun Li, Changhua Hu, Junping Yao, Xiaoxia Han, Jie Wang

**Affiliations:** High-Tech Institute of Xi’an, Xi’an, 710025 Shaanxi China

**Keywords:** Information technology, Computer science

## Abstract

Spammer detection is essentially a process of judging the authenticity of users, and thus can be regarded as a classification problem. In order to improve the classification performance, multi-classifier information fusion is usually used to realize the automatic detection of spammers by utilizing the information from multiple classifiers. However, the existing fusion strategies do not reasonably take the uncertainty from the results of different classifiers (views) into account, and the relative importance and reliability of each classifier are not strictly distinguished. Therefore, in order to detect spammers effectively, this paper develops a novel multi-classifier information fusion model based on the evidential reasoning (ER) rule. Firstly, according to the user's characterization strategy, the base classifiers are constructed through the profile-based, content-based and behavior-based. Then, the idea of multi-classifier fusion is combined with the ER rule, and the results of base classifiers are aggregated by considering their weights and reliabilities. Extensive experimental results on the real-world dataset verify the effectiveness of the proposed model.

## Introduction

With the rapid development of the internet and big data technology, social media has gradually become important. Unfortunately, while enabling people to communicate with each other more conveniently, it also offers a hotbed for a large number of spammers. A spammer is a user who uses social media platforms to spread malicious information such as false information and inappropriate comments, which poses serious security risks to people’s daily lives^1^. Therefore, it has become an urgent issue to study how to detect spammers from numerous social media accounts effectively.

However, spammers keep updating their communication tactics to avoid being tracked with the fast development of relevant technologies. Therefore, it is impossible to realize comprehensive and real-time modeling and detection of spammers from a single perspective^2^. Some scholars considered adopting a multi-classifier information fusion approach to solve this problem. Chen et al.^3^ proposed a novel approach called semi-supervised clue fusion (SSCF), which acquires a linear weighted function to fuse the comprehensive clues explored from multiple aspects to obtain final results effectively. Fazil et al.^4^ found that spammers can bypass detection systems by avoiding features related to individuals. They proposed a hybrid model for detecting automated spammers with a better generalization ability by amalgamating community-based features with other feature categories, namely metadata-based, content-based, and interaction-based features, and sorting out six newly-defined features. Yin et al.^5^ attempted to fully exploit the sequences of heterogeneous relations based on the personal and social features of users, and proposed the multi-level dependency model (MDM), whose effectiveness was demonstrated by the experimental results on a multi-relational social network. Liu et al.^6^ proposed a novel modeling scheme that combined user behavior, information content, and social network (such as Follow and Repost), and introduced the crowdsourcing mechanism to realize the cooperative detection of spammers.

Reviewing the above literatures, it can be found that the effect of multi-classifier information fusion depends largely on the fusion method selected. However, the existing fusion strategies do not well represent and combine the uncertainty of results from different classifiers (views), and they tend to provide unreliable classification results when a single classifier (view) cannot render a good representation^7^. In addition, the traditional algorithms often focus on using fixed aggregation strategies for classification purposes, which either do not consider the relative importance and reliability of each classifier, or do not strictly distinguish the two concepts. In practical applications, different feature views may be adapted to different samples. Thus, it is necessary to propose a multi-classifier information fusion model with an adaptive fusion strategy.

As a widely-used approach to information fusion, the evidential reasoning (ER) rule boasts a strong ability to process and analyze multi-source information uncertainty^8^. The overall performance of the system can be improved by training a classifier for each view and combining the ER rule with the multi-classifier ensemble. In addition, the parameters of the ER rule, such as reliability and weight, can be used to express the internal and external features of the multi-classifier system simultaneously, thus enhancing the interpretability of the system. Although the multi-classifier fusion based on the ER rule has proved to be a highly promising method in many applications^9,10^, relevant research showed that this method is still faced with the following challenges. (1) The ER rule, as a general form of the traditional Bayes method, is built on multiple pieces of independent evidence. Therefore, the first challenge is how to combine a set of base classifiers with the ER rule and build a reasonable multi-classifier model based on the ER rule. (2) Reliability and weight are regarded as two important parameters of the ER rule and are used to represent the objective attribute and subjective attribute of evidence, respectively. The second challenge is how to use these parameters to properly express the model in a multi-classifier system and ensure that it achieves a better generalization ability and interpretability.

To overcome these challenges, we attempt to develop a new spammer detection method that can identify spammers under various complicated factors, including uncertainty, interference and even deviation. Specifically, the innovations of our work mainly include the following two aspects:

1. A novel multi-classifier information fusion model is developed, aiming to detect the spammers present in social networks in an efficient and effective manner. In this model, the characteristics of spammers are divided into multiple views, and the machine learning method is used to learn the knowledge of each view, so as to use the idea of multi-classifier fusion to achieve effective detection of spammers;
2. This work elegantly combines the idea of multi-classifier fusion with the ER rule. By giving a new acquisition process of weight and reliability to the base classifier, it can dynamically integrate the uncertain information from different views at the evidence level, which provides a new paradigm for the multi-classifier fusion. On this basis, through extensive experiments, the excellent accuracy and robustness of our model are verified.

The rest of this paper is as follows. Chapter 2 is the Model framework and problem formulation part, which introduces the brief framework of the model proposed in this paper and the two core problems (challenges) to be solved by this work. In order to solve the problem mentioned above, in Chapter 3, we introduced the methods used in this paper, including the base classifier generation, the calculation method of reliability and weight, and the multi-classifier information fusion based on the ER rule. Chapter 4 is the Case study part, which adopts the method introduced in Chapter 3, including data preprocessing, experimental design, parameter setting and result analysis. By comparing the proposed method and other methods and analyzing the experimental results, the effectiveness of our method was validated. In Chapter 5, we made a conclusion and discussed some work to be explored in the future.

## Model framework and problem formulation

**Figure 1:**
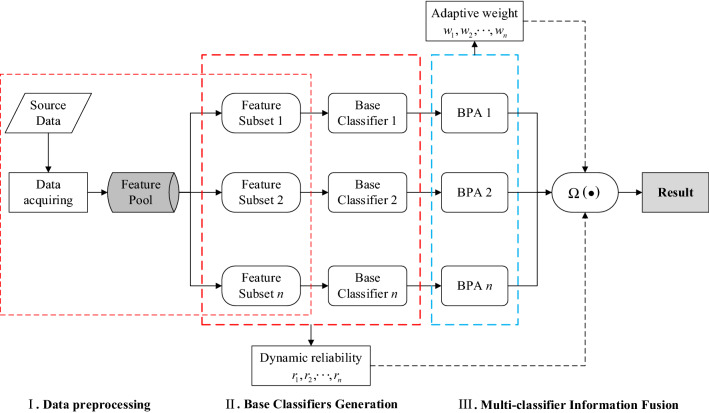
The structure of the proposed model.

Figure 1 shows the model constructed in this paper based on actual problems, where $\Omega ( \bullet )$ represents a nonlinear function, which is also a computing framework for multi-classifier information fusion.

As shown in Fig. 1, the model mainly consists of three parts. The first part is data preprocessing. By acquiring data samples needed from the source data, a feature index system was built, and these feature indexes constituted a feature pool. The feature pool could be split into multiple feature subsets comprised of comprehensive information. Each feature subset contains a number of attributes. The second part is the base classifier generation. A base classifier was used to classify the views grouped above. Since it is difficult for most classifiers to obtain accurate category probabilities, a specific transformation method needs to be introduced to make the final basic probability assignment (BPA) more competitive^11^. In the third part, an effective fusion strategy is used to conduct multi-classifier information fusion. It is noted that the performance, internal attributes and relative importance of classification results of each classifier should be considered during fusion, which has a great impact on the final decision^12^.

According to the model architecture built in Fig. 1, the following two problems should be considered to improve the overall performance of the multi-classifier system and thus detect spammers in a more targeted way in actual applications.

Problem 1: To obtain comprehensive detection results from different characteristic views, all view-related information must be considered comprehensively. Meanwhile, the model needs to combine the internal attributes and the relative importance of multiple views to realize adaptive fusion. Therefore, Problem 1 is focused on building the framework of multi-classifier information fusion as follows:

1 $$R = \Omega ( \cdot )$$

where $\Omega ( \cdot )$ represents a nonlinear function.

Problem 2: Because different data samples are subject to dynamic change, the observed feature subsets of spammers will have a varying influence on the results, which further leads to the relative difference between base classifiers and the classification uncertainty of these classifiers. Therefore, these factors should be considered separately during fusion, as shown in Eq. (2).

2 $$\begin{gathered} w_{i} = f(I(v_{1} ),I(v_{2} ), \ldots ,I(v_{3} )) \, \hfill \\ r_{i} = g(I(v_{1} ),I(v_{2} ), \ldots ,I(v_{3} )) \hfill \\ \end{gathered}$$

where $f( \cdot ),g( \cdot )$ represent nonlinear functions and $I(v_{n} )$ means the classification information of the corresponding $n{\text{th}}$ feature subset classifier. Therefore, to obtain better detection results while endowing the system with interpretability, Problem 2 mainly involves how to obtain these important parameters through reasonable calculation.

## Methodology

**Figure 2:**
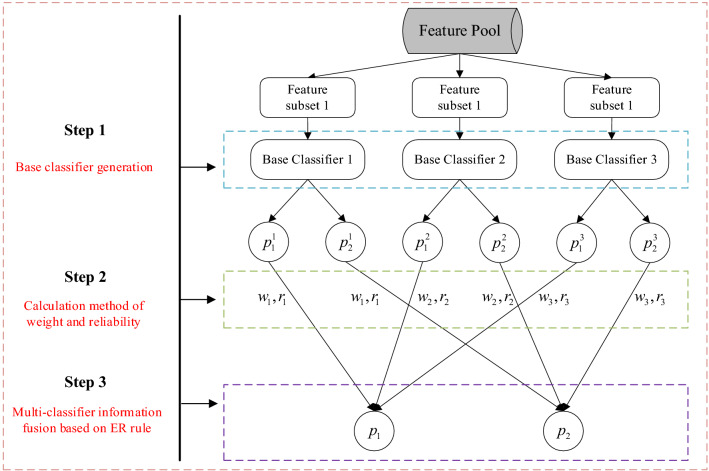
The implementation procedure of the proposed model.

As shown in Fig. 2, the implementation process of the proposed model is mainly introduced in this part, including the base classifier generation, the calculation method of weight and reliability, and multi-classifier information fusion based on the ER rule. $p_{j}^{m}$ represents the belief degree by the $m$th classifier to the $j$th category, and $p_{j}$ represents the belief degree by the system to the $j$th category after fusion. The rest of this chapter will elaborate on the above three steps.

### Base classifier selection

Unlike the traditional methods that only need to train one classifier, multi-classifier information fusion needs to train and generate multiple classifiers at the same time, and then combine them to solve practical problems^13^. Therefore, choosing a suitable base classifier as the fusion material is crucial for improving the performance of the multi-classifier system. So far, a large number of classification models have been proposed and widely used in various fields^14^, among which K-Nearest Neighbors (KNN), Support Vector Machines (SVM) and Artificial Neural Networks (ANN) are representative. The advantage of KNN is that the modeling is simple and does not require parameter estimation and training process. However, the size of its k value needs to be determined manually, and it is very sensitive to class imbalanced data^15^. Therefore, it is not suitable for use as a base classifier in this study. ANN establishes a mapping relationship between attribute input and output by simulating the process of human thinking. It is considered to be a promising classification technology with high accuracy. However, it still has some inevitable disadvantages, such as process black box, easy overfitting, and feature dimensionality curse^16^. In contrast, SVM has the following significant advantages^16,17^: (1) SVM has excellent prediction and generalization ability; (2) There is no strict requirement for feature dimension; (3) Overfitting problem is solved by dividing unique decision boundary surface; (4) The model structure is stable. Therefore, this research will choose SVM as the base classifier of our model.

The multi-classifier information fusion based on ER rule is essentially a soft computing process. To solve the problem that the traditional SVM cannot output classification belief degree, this article will use the Platt scaling method, based on the sigmoid function, as shown in Eq. (3). The output of SVM can be mapped into a posterior probability which ranges from 0 to 1^18^.

3 $$P(y = 1|s(x)) = \frac{1}{1 + \exp (As(x) + B)}$$

where $x$ is the input, and $s(x)$ is the non-threshold output of SVM. $A$ and $B$ represent the two parameters in the maximum likelihood estimation training, and the objective function is:

4 $$\min - \sum\limits_{i} {\frac{{y_{i} + 1}}{2}\log (P(x_{i} )) + \left( {1 - \frac{{y_{i} + 1}}{2}} \right)} \log (1 - P(x_{i} ))$$

where $P(x_{i} )$ = $P(y = 1|s(x))$, representing a posterior probability.

### Calculation method of weight and reliability

The fusion quality of the ER rule is generally determined by the calculation results of weight and reliability, and these parameters are also the prerequisite to distinguish the ER Rule from other methods. An example was used to illustrate how the ER rule changes the final decision by adjusting such parameters as weight and reliability in the multi-classifier ensemble.

#### Example 1

Suppose there are three base classifiers {*C*_1_; *C*_2_; *C*_3_} whose preliminary classification results are {(0.72, 0.28); (0.31, 0.69); (0.56, 0.44)} and only *C*_*2*_ is correct. If we assigned the same weight and reliability {(0.5, 0.5); (0.5, 0.5); (0.5, 0.5)} to each base classifier, the belief degree after fusion would be assigned with {(0.5432, 0.4568)}. According to common sense, the classifier that can correctly classify should be assigned higher belief degree, namely Category I. It can be seen that the method assigned with the same weight and reliability did not change the final decision category. However, if we increased the weight and reliability of *C*_2_ and decreased the weight and reliability of *C*_1_ and *C*_3_, then the weight and reliability of the three base classifiers were assigned with {(0.3, 0.4); (0.8, 0.7); (0.4, 0.3)} and the final calculated belief degree distribution was {(0.4276,0.5724)}. This example showed that the misclassification of a classifier could be corrected by adjusting the weight and reliability of the ER rule based on the actual situation.

Clearly, it is vital to define weight and reliability. In the ER rule, weight $w_{i}$ is regarded as the degree of preference of a decision-maker to the $i$th evidence item, and reliability $r_{i}$ is seen as the internal attribute of the information source generating the $i$th evidence item^19^, where $i$ represents the $i$th training sample ($i = 1,2, \cdot \cdot \cdot ,N$). They correspond to the subjective attribute and objective attribute of evidence, respectively. The base classifiers in the multi-classifier system all learn based on different attribute knowledge, so they may have different classification abilities. Generally, the base classifier model which performs better can have a greater effect on the results, so it should be assigned with a higher weight and higher reliability^20^. On this basis, in this paper, weight and reliability were preliminarily defined as the relative difference between base classifiers and the classification uncertainty of these classifiers.

First, a new weight calculation method based on probability distance was proposed. This method can be used to express the external difference between different classifiers. Dice coefficient is regarded as an effective function for measuring probability similarity, which is usually used to calculate the similarity between different sample elements^21^. Its definition is shown in Eq. (7):

5 $$Dice = \frac{{2\left| {X \cap Y} \right|}}{\left| X \right| + \left| Y \right|}$$

where $X$ and $Y$ represent the set of elements in the two samples respectively, and they are expressed as $X \cap Y$. Dice coefficient was introduced into the multi-classifier ensemble system. In this paper, the similarity between the belief degree by a classifier and the average belief degree by all classifiers in a training sample is expressed as follows:

6 $$SIM_{m} = \frac{{2\sum\nolimits_{j = 1}^{C} {\sum\nolimits_{i = 1}^{N} {p_{ij}^{m} \overline{{p_{ij} }} } } }}{{\sum\nolimits_{j = 1}^{C} {\sum\nolimits_{i = 1}^{N} {(p_{ij}^{m} )^{2} } } + \sum\nolimits_{j = 1}^{C} {\sum\nolimits_{i = 1}^{N} {(\overline{{p_{ij} }} )^{2} } } }}$$

where $m$ represents the $m$th base classifier ($m = 1,2, \cdot \cdot \cdot ,L$), $j$ represents the $j$th category ($j = 1,2, \cdot \cdot \cdot ,C$), $p_{ij}^{m}$ denotes the belief degree by the $m$th classifier to the $j$th category of the $i$th training sample, and $\sum\nolimits_{j = 1}^{C} {p_{ij}^{m} } = 1$. $\overline{{p_{ij} }}$ is the average belief degree by the $i$th training sample to the $j$th category. Therefore, the diversity of the $m$th classifier was defined in Eq. (9) through perfect square calculation.

7 $$\begin{aligned} DIV_{m} = 1 - SIM_{m} & = \frac{{\sum\nolimits_{j = 1}^{C} {\sum\nolimits_{i = 1}^{N} {(p_{ij}^{m} )^{2} } } + \sum\nolimits_{j = 1}^{C} {\sum\nolimits_{i = 1}^{N} {(\overline{{p_{ij} }} )^{2} } } - 2\sum\nolimits_{j = 1}^{C} {\sum\nolimits_{i = 1}^{N} {p_{ij}^{m} \overline{{p_{ij} }} } } }}{{\sum\nolimits_{j = 1}^{C} {\sum\nolimits_{i = 1}^{N} {(p_{ij}^{m} )^{2} } } + \sum\nolimits_{j = 1}^{C} {\sum\nolimits_{i = 1}^{N} {(\overline{{p_{ij} }} )^{2} } } }} \hfill \\ & = \frac{{\sum\nolimits_{j = 1}^{C} {\sum\nolimits_{i = 1}^{N} {(p_{ij}^{m} - \overline{{p_{ij} }} )^{2} } } }}{{\sum\nolimits_{j = 1}^{C} {\sum\nolimits_{i = 1}^{N} {(p_{ij}^{m} )^{2} } } + \sum\nolimits_{j = 1}^{C} {\sum\nolimits_{i = 1}^{N} {(\overline{{p_{ij} }} )^{2} } } }} \hfill \\ \end{aligned}$$

For richer diversity of multi-classifier ensemble, the greater contribution of a base classifier to the diversity implies that the classifier can provide more effective information than others during classification^22^. Therefore, if the diversity of a single classifier is stronger, it should be given a higher weight. The weight of the $m$th classifier is defined as:

8 $$w_{m} = \frac{{DIV_{m} }}{{\sum\nolimits_{m = 1}^{L} {DIV_{m} } }}$$

In the proposed model, reliability is regarded as an index that can measure the internal classification uncertainty of a base classifier. In this paper, reliability calculation is mainly based on the inherent error of the classifier and the ability of the input sample to identify each mode, both of which can reflect the overall classification performance of the classifier. In the case of spammer detection, we hope to minimize the misclassification of important users. As to such a cost-sensitive classification issue, if inappropriate evaluation indexes are used, which covers up the fact that the samples are misclassified, the classification performance of the classifier cannot be correctly reflected^23^. The area under the curve (AUC) is defined as the area enclosed by the coordinate axis underneath the receiver operating characteristic (ROC) curve, representing the probability that a predicted target class is ranked before a non-target class. A higher AUC value indicates that the classifier is more likely to rank the real target class sample before others, and the classification performance is better. Hence, it is regarded as an excellent evaluation index to approach cost-sensitive problems. In this paper, the AUC value of a single base classifier on the validation set is taken as the reliability of the classifier, as shown in Eq. (16):

9 $$r_{m} = AUC_{m}$$

It can be seen that the weight and reliability of each classifier are dynamically represented and are determined adaptively according to the quality of the base classifier trained by the sample data, without requiring prior knowledge about the dataset.

### Multi-classifier information fusion based on the ER rule

Multi-classifier ensemble has proved to be a fault-tolerant method with an excellent classification ability in wide practical applications^24^. An excellent ensemble method can effectively fuse the base classifier-related information based on different feature subsets. With the aid of the ensemble strategy, it can transform the predicted information from different classifiers into necessary decision information. In other words, the final decision results are directly determined by the quality of the ensemble strategy. However, we found that many previous studies still focused on the ensemble based on a weighted average and majority voting and failed to update relevant algorithms actively^25,26^. As mentioned above, spammers are good at disguising their behaviors to avoid being detected by artificial algorithms, making it challenging to obtain satisfying results through the previous methods. Furthermore, the correlation between base classifiers and their classification characteristics should be considered comprehensively during fusion, which is unavailable in previous methods. In this paper, SDMER is proposed to cope with the real-time update of spammers’ detection avoidance tactics in the increasingly complex detection environment and realize an overall detection structure that can make up for the shortcomings of existing ensemble strategies. Built on Dempster Shafer’s evidence theory, the ER rule introduces parameters such as weight and reliability, which has greatly enhanced the human–machine capability and interpretability of the system^8^ and improved the classification and decision-making performance. It has been extensively researched and applied in medical image, fault diagnosis, individual credit evaluation and other fields^12,27,28^.

The evidence item of the ER rule refers to the belief degree distribution of evidence, which is generally derived from prior knowledge or expert system. In the ER rule, it is assumed that the frame of discernment (FOD) is $\Theta = \{ \beta_{n} \left| {n = 1, \cdot \cdot \cdot ,T} \right.\}$, where $n$ represents the $n$th evaluation. It can be viewed as the category in the classification problem and also a set of mutually exclusive and collectively exhaustive propositions.

10 $$M(\Theta ) = \left\{ {\emptyset ,\beta_{1} ,\beta_{2} , \ldots ,\beta_{n} ,\left\{ {\beta_{1} ,\beta_{2} } \right\}, \ldots ,\left\{ {\beta_{1} ,\beta_{2} , \ldots ,\beta_{n} } \right\},\Theta } \right\}$$

These evidence items are the category probability output from each base classifier in a multi-classifier ensemble system. A piece of evidence can be described with the belief degree distribution as follows.

11 $$e_{i} = \{ (\beta_{n} ,p_{{\beta_{n} ,m}} ),\forall \beta_{n} \subseteq \Theta ,\sum\nolimits_{n = 1}^{T} {p_{{\beta_{n} ,m}} = 1} \}$$

where $p_{{\beta_{n} ,m}}$ represents the belief degree of the $m$th classifier to the category $\beta_{n}$, and meets $0 \le p_{{\beta_{n} ,m}} \le 1$. The weighted belief degree distribution of evidence with the addition of reliability can be expressed as $\tilde{\varepsilon }_{{\beta_{n} ,m}}$ and is defined as follows.

12 $$\tilde{\varepsilon }_{{\beta_{n} ,m}} = \left\{ \begin{gathered} {0,}\,\,\,\,\,\,\,\,\,\,\,\,\,\,\,\,\,\,\,\,\,\,\,\,\,\,\,\,\,\,\,\,\,\,\,\,\beta_{n} = \emptyset \hfill \\ \mu_{rw,m} \varepsilon_{{\beta_{n} ,m}} ,\,\,\,\,\,\,\,\,\,\,\,\beta_{n} \subseteq \Theta ,\beta \ne \emptyset \hfill \\ \mu_{rw,m} (1 - r_{m} ),\,\beta_{n} = M\left( \Theta \right) \hfill \\ \end{gathered} \right.$$

where $\emptyset$ represents the empty set, $\varepsilon_{{\beta_{n} ,m}} = w_{m} p_{{\beta_{n} ,m}}$ and $\mu_{rw,m} = 1/(1 + w_{m} - r_{m} )$.

It is noted that the ER rule has proved to be a generalized concept of Bayes’ Rule, which means that evidence should be independent and mutually exclusive^8^. Under this premise, classification in this paper was conducted in the method during feature selection and was divided into multiple views according to this criterion. Therefore, by default, the evidence output by each classifier was independent of each other. For any pair of independent evidence, the joint belief degree of the proposition can be expressed as $p_{{\beta_{n} }} (x)$^8,12^, which can be calculated through the ER rule:

13 $$p_{{\beta_{n} }} (x) = \frac{{K\left[ {\prod\nolimits_{m = 1}^{L} {\left( {\frac{{1 - r_{m} }}{{1 + w_{m} - r_{m} }} + \frac{{w_{m} p_{{\beta_{n} ,m}} }}{{1 + w_{m} - r_{m} }}} \right)} - \prod\nolimits_{m = 1}^{L} {\left( {\frac{{1 - r_{m} }}{{1 + w_{m} - r_{m} }}} \right)} } \right]}}{{1 - K\prod\nolimits_{m = 1}^{L} {\left( {\frac{{1 - r_{m} }}{{1 + w_{m} - r_{m} }}} \right)} }}$$

where $K$ represents the normalized coefficient, which is calculated as follows:

14 $$K = \left[ {\sum\nolimits_{j = 1}^{C} {\left( {\prod\nolimits_{m = 1}^{L} {\left( {\frac{{1 - r_{m} }}{{1 + w_{m} - r_{m} }} + + \frac{{w_{m} p_{{\beta_{n} ,m}} }}{{1 + w_{m} - r_{m} }}} \right)} } \right)} - (C - 1)\prod\nolimits_{m = 1}^{L} {\left( {\frac{{1 - r_{m} }}{{1 + w_{m} - r_{m} }}} \right)} } \right]^{ - 1}$$

Moreover, the above procedure was performed for all samples.

## Case study

### Case background

The data in this study were collected from one of China’s popular social media platforms by professionals. After a certain quantity of data samples were collected and preprocessed, those repeated and redundant users and features were filtered, and volunteers and experts were invited to annotate the dataset. It is worth mentioning that the number of legitimate users and spammers was balanced without reducing the validity of the research results which means there are 550 legitimate users and 550 spammers, resulting in a user characteristic dataset with a sample size of 1,100. All methods were carried out in accordance with relevant guidelines and regulations.

**Figure 3:**
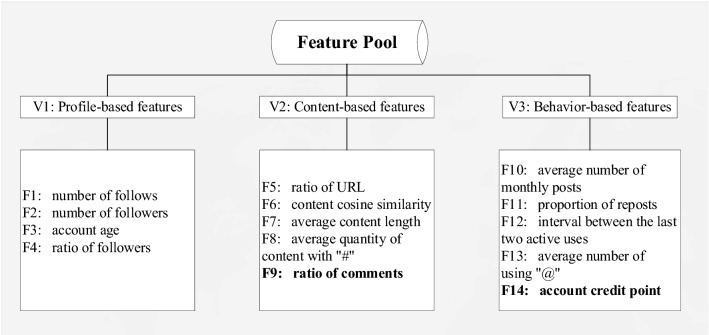
Development of a framework for feature grouping.

User feature extraction is of great importance to spammer detection. Due to the complexity of social media networks, user features are characterized by high dimensions and redundancy. As shown in Fig. 3, considering that there are many factors affecting the spammer detection environment, the feature attributes of social network users were divided into the following three different views based on the research status in China and abroad.

1. Profile-based features (V1): First, spammers have a much lower number of followers (F1)^30^ than legitimate users because spammers do not rely on a large number of followers to spread false information or spam information. The second feature is the number of followers (F2)^29^. Studies showed that the number of followers of spammers is also significantly different from that of legitimate users because most of these accounts do not engage in normal social relations. The third feature is account age (F3)^30^. To spread rumors widely and avoid detection, the account age of spammers is relatively low. The fourth feature is the ratio of following to followers (F4)^30^. There is a remarkable difference in the ratio of following to followers between spammers and legitimate users.
2. Content-based features (V2): The first content-based feature is the ratio of URL (F5)^31^. The content posted by spammers involves more malicious links. The second feature is content cosine similarity (F6)^32^, which mainly records the cosine similarity of the two newly-posted pieces of content. Spammers tend to post a high proportion of the same content in a short period. The third feature is the average content length (F7)^30^. The average length of content posted by spammers is higher than that of legitimate users. The fourth feature is the average quantity of content with "#" (F8)^33^, and spammers use more tags in the content posted. The fifth feature is the ratio of content to comments (F9), which is a content-based feature newly proposed in this study. It records the ratio of original content to received comments. Legitimate users interact with their online friends normally, while spammers rarely receive comments due to the low value of their content.
3. Behavior-based feature (V3): The first feature is the average number of monthly posts (F10)^6^. Spammers may engage in short-term illegal activities for profit, which indicates that their average number of monthly posts is not too high. The second feature is the proportion of reposts in all posts (F11)^29^. Spammers are more inclined to repost and thus spread illegal information more widely. The third feature is the interval between the last two active uses of an account (F12)^33^. Compared with legitimate users, spammers tend to be less active. The fourth feature is the average number of using "*@*" (F13)^31^. Spammers are more likely to mention (or "@") other users to attract greater attention. The fifth feature is the account credit point (F14), which is a new feature proposed in this paper. The account credit is measured with a user's daily performance recorded by this platform. It was mapped as an integer value between 1 and 5 in this study.

### Experimental design and parameter setting

**Figure 4:**
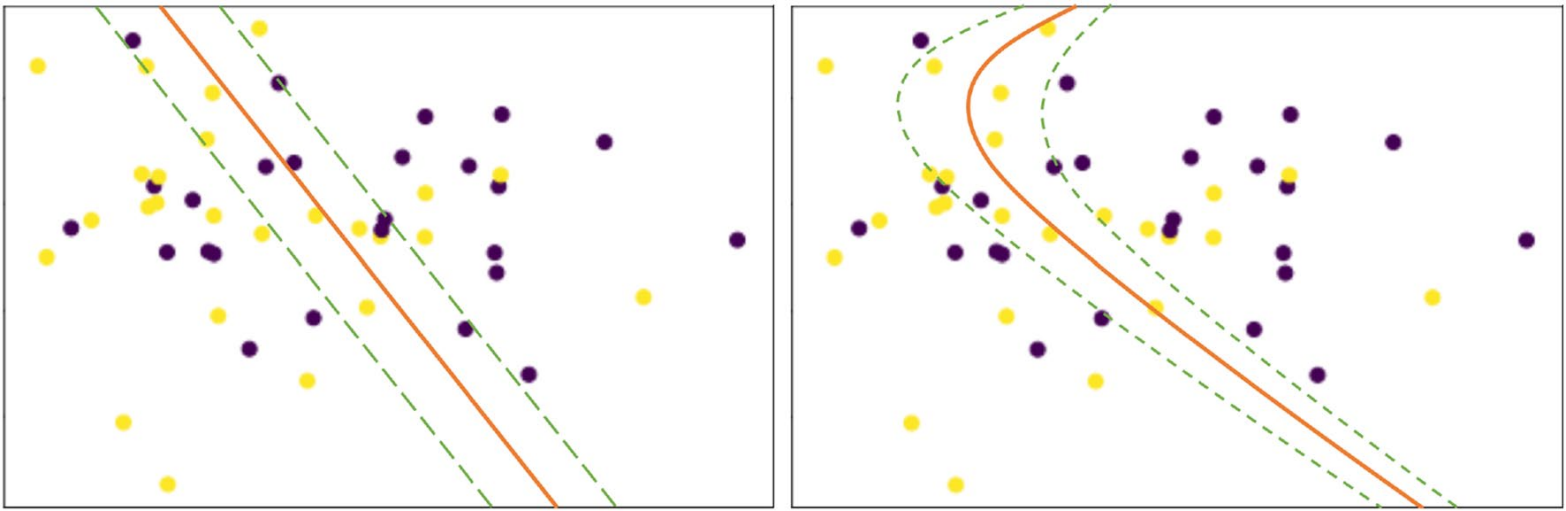
SVM classification results based on linear kernel and polynomial kernel functions.

This section mainly introduced the design of the experimental process and several parameters selected in this study. The parameters that affect the classification performance of SVM include the selection of kernel function, parameters of kernel function and penalty factor $C$. The separation effect of maximal margin hyperplane on feature space is generally determined by the setting of different parameters. Any pair of content-based features were used to draw a scatter plot. As shown in Fig. 4, when a linear kernel function or a polynomial kernel function was selected, its decision boundary did not have the desired separation effect on the two categories of scatter points, which may be due to factors such as overlap and chaos between scattering points. RBF is known as an effective method to tackle linear inseparability and display excellent classification performance, especially when feature samples are mapped to high-dimensional space^34^. Therefore, SVM based on RBF was adopted in this study, and the parameters to be optimized were kernel function parameter $\gamma$ and penalty factor $C$.

GridSearchCV was adopted in this study to optimize parameters and find the optimal combination of $\gamma$ and $C$. The advantage of this method is that it can traverse all possible parameter combinations to find a group of parameters that meet the optimization requirements. Moreover, this method is also highly adaptable to small sample data^35^. On this basis, tenfold cross-validation was conducted on the data of each base classifier. The benefits of this method are involving as much data as possible in training and testing, minimizing the classification error through interactions for average, and obtaining the best parameter optimization results. The combination of parameters selected by the SVM of each base classifier is shown in Table 1, corresponding to the optimal accuracy of their respective models.

**Table 1:** The parameter optimization results of the base classifier and accuracy.

Base classifier	SVM 1	SVM 2	SVM 3
($\gamma$, $C$)	(1100, 0.8)	(1100, 5)	(1000, 0.9)
Accuracy	83.92%	84.43%	83.13%

**Figure 5:**
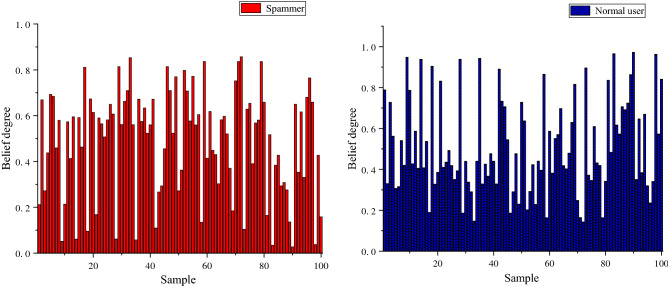
Belief degree from classifier of behavior-based features.

After the optimal SVM classifier corresponding to each view was obtained, the method in “Base classifier selection” section was used to transform the results of the classifier output into a posterior probability, which is the belief distribution needed for the ER rule input. Given the limited space, Fig. 5 shows part of the belief degrees from the corresponding test samples of the classifier based on behavior features. Among them, the horizontal axis represents the count of samples, and the vertical axis represents the belief degree of the classifier corresponding to the test sample. It is worth noting that the value range of the belief degree is $\left[ {0,1} \right]$. In addition, the red bars represent the belief degree that these test samples are recognized as spammers by the classifier, and the blue bars represent the belief degree that the test samples are recognized as normal users.

To validate the effectiveness of the proposed SDMER method, we compared it with other multi-classifier fusion methods in our experiment. Several common multi-classifier fusion methods applied at the decision level include soft-voting (SV), weighted soft-voting (WSV), Dempster-Shafer evidence theory (DS), and the evidential reasoning algorithm (ERA). It is noted that we adopted the trained SVM in SDMER for all base classifiers and used the same values as those in SDMER for the weight calculation method of WSV and ERA, so as to draw more accurate comparison results. To ensure that the comparison results are more general, we also added several common ensemble learning methods, including Bagging, AdaBoost, Random Forest (RF) and eXtreme Gradient Boosting (XGBoost)^36^, to the comparative study. The setting of some important parameters during operation is shown in Table 2.

**Table 2:** The parameter setting of the ensemble learning methods.

Methods	Parameters
Bagging	Number of ensembles: 10; Base classifier: SVM; Number of max samples: 1.0; Number of max features: 1.0; Bootstrap: True
AdaBoost	Number of ensembles: 10; Base classifier: SVM; Algorithm: ‘SAMME.R’; Learning rate: 1.0
Random forest	Number of ensembles: 100; Criterion: Gini; Max depth: None; Min samples split: 2; Min samples leaf: 1; Bootstrap: True
XGBoost	Number of ensembles: 100; Max depth: 6; Gamma: 2; Min child weight: 1; Learning rate: 0.3; Subsample: 1

After referring to relevant research, two recently released solutions are also added in this work, Gaussian Naive Bayesian (GNB)^37^ and a new multi-classification credit assessment model (MIFCA)^38^. Among them, GNB is a novel classification method for detecting illegal uploaders in social media, and MICFA is a new method based on multi-classifier information fusion. This work selected accuracy, precision, recall and F1 score to evaluate the performance of the proposed SDMER model and focused on analyzing its spammer detection effect. Accuracy, precision and recall are usually used to evaluate the performance of classification in machine learning, while the F1 score can better reflect the comprehensive performance of the model^39^. These indexes are defined as follows:

Accuracy refers to the proportion of correct classification in all classifications and is defined as follows.

15 $$Accuracy = \frac{TP + TN}{{TP + TN + FP + FN}}$$

where *TP* represents the number of spammers correctly classified, *TN* means the number of legitimate users effectively detected, *FP* denotes the number of legitimate users wrongly classified as spammers, and *FN* stands for the number of spammers wrongly classified as legitimate users.

Precision is the proportion of real spammers to all spammers classified and is defined as:

16 $$Precision = \frac{TP}{{TP + FP}}$$

Recall is the proportion of spammers effectively detected in all spammers, and is defined as:

17 $$Recall = \frac{TP}{{TP + FN}}$$

F1 score represents the harmonic mean between precision and recall, which is defined as:

18 $$F1 - score = \frac{2Precision}{{Precision + Recall}}$$

**Figure 6:**
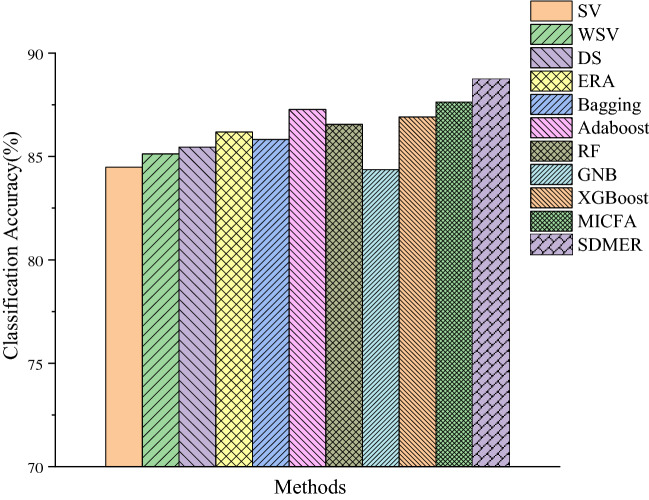
Comparison of classification accuracy in all methods.

The comparison of accuracy between these models is shown in Fig. 6. All the final results are presented in Table 3.

**Table 3:** Comparison of classification performance of various methods.

Methods	Accuracy	Precision	Recall	F1-score
SV	84.48%	88.32%	81.76%	0.8493
WSV	85.12%	**90.51%**	81.58%	0.8582
DS	85.45%	89.05%	82.99%	0.8589
ERA	86.18%	87.22%	84.67%	0.8593
Bagging	85.82%	85.40%	86.03%	0.8571
AdaBoost	87.27%	89.05%	85.92%	0.8737
Random forest	86.55%	84.67%	87.88%	0.8625
GNB	84.36%	85.07%	83.21%	0.8413
XGBoost	86.91%	85.82%	87.12%	0.8647
MICFA	87.63%	87.05%	88.32%	0.8768
SDMER	**88.73%**	87.59%	**89.55%**	**0.8856**

Significant values are in [bold].

**Figure 7:**
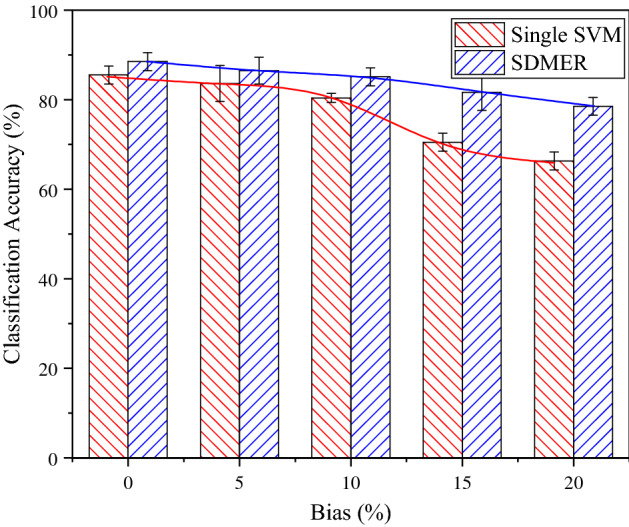
Comparison of classification accuracy in the Single SVM model and the SDMER model.

There is no doubt that we cannot find almost perfect experimental data in real life and the environment for spammer detection is special. Therefore, the proposed model must have the anti-interference or anti-deviation capability. To validate the performance of the model with data deviation, we described the change of classification accuracy of the proposed method with different levels of deviation in the source data (5, 10, 15 and 20% of noise interference are added to the training sample) in Fig. 7.

### Result analysis

The results above are further discussed. (1) As shown in Table 3, according to the comparison between the actual operation results and the performance of each baseline, the proposed SDMER model outperformed other methods in terms of accuracy, recall and F1 score. Although its precision was slightly lower than some other methods, it still fell within an acceptable range. Among all the methods based on decision-level fusion, the precision of these models was at a relatively high level, which is explained as follows. First, after pre-training and parameter optimization, the SVM model showed an excellent learning effect on data samples, which was reflected in the accurate classification and posterior probability in the training and testing process, namely the effective belief degree. Second, the excellent performance represented the sensitivity of the model in detecting spammers, which further validated the rationality and effectiveness of the model framework proposed in “Case background” section. It is interesting to find that the overall performance of the proposed SDEMR model was slightly better than that of ERA, which indicates that the introduction of reliability had a positive effect on the improvement of overall performance. It is noted that accidental factors cannot be ignored in practice. Besides comparing the two categories of models, the effect of reliability on model performance will be further researched in the future.

(2) As shown in Fig. 7, given the sample deviation of 5%, the classification accuracy of the SVM model alone and the proposed SDMER model was subject to a low level of interference, and there was no obvious difference. When the deviation rose to more than 10%, the SVM model alone showed obvious fluctuation, and its accuracy was on the decrease. Especially when the deviation exceeded 20%, the accuracy of the SVM model alone declined to a relatively low level (below 70%). Although the accuracy of the SDMER model also declined in varying degrees, it could still be close to 80%. In other words, the SVM model alone showed high sensitivity to deviation, and its accuracy decreased significantly with the increase in deviation. By contrast, the SDMER model was much less sensitive to deviation. According to analysis results, as the data sample changed, the SDMER model could fully utilize all the information of the ensemble-based classifier and adjust the parameters such as weight and reliability in an adaptive manner to make classifiers complementary to each other. As a result, the classification performance of the whole system would not be affected negatively and obviously. However, it is well known that most machine learning methods are sensitive to data and parameters, and their classification performance will be significantly reduced when the sample data deviates or changes. As mentioned in the Introduction, the spammer detection environment has a high level of uncertainty and complexity. Therefore, the proposed SDMER model is an effective tool to solve this problem.

## Conclusions and future works

In this work, we propose a unified framework called spammer detection using multi-classifier information fusion based on evidential reasoning rule (SDMER), this work aims to combine multi-classifier information fusion with the improved ER rule, a new spammer detection method is proposed to provide a more comprehensive and accurate detection effect. Overall, the proposed method involves three main stages: (1) We select and train classifiers corresponding to different feature views of spammers in a reasonable way, and convert the classification results into the form of belief degree distribution; (2) A new calculation method is proposed for the importance weight factor and reliability factor respectively, so that it can better distinguish and express subjective uncertainty and objective uncertainty; (3) Use ER rule to fuse the obtained belief degree distribution information from each classifier at the decision-making layer, and finally obtain the overall classification result of the system.

The main contributions of this paper are as follows: the first important contribution is to develop a novel multi classifier information fusion model to effectively detect spam users in social networks. In our method, the spammer features in social networks are divided into multiple views, and the machine learning method is used to train the information from each view, and then the final results are obtained by multi classifier fusion. Another significant contribution is to fully combine the idea of multi-classifier information fusion and the advantages of ER rule. By giving the base classifier a new acquisition process of weight and reliability, it can dynamically integrate the uncertain information from different views at the evidence level. Sufficient comparative studies show that SDMER can obtain better accuracy and stability on the basis of the above.

It is worth pointing out that although the SDMER model proposed in this paper has been verified to have good results in our experiments, there is still a lot of work to be solved in the future. In terms of spammer detection, it is noted that all experimental results in this paper only apply to the real-world dataset in this study. Currently, much research on exploring and modeling spammer features remains to be done, which presents a great challenge to spammer detection and even feature engineering in the field of false information detection. Additionally, in terms of multi-classifier information fusion, only the isomorphic base classifier is considered in our proposed model, but in theory, more types of advanced classifiers can participate in the process of multi-classifier information fusion, such as deep learning-based classifiers and other multi-task learning-based classifiers, it is an interesting work to study how to introduce more types of classifiers into SDMER. Moreover, further research work must be done on how to obtain and optimize weight and reliability, which are two important parameters of SDMER, in new ways.

## Data Availability

The datasets generated and/or analysed during the current study are not publicly available due to involving the common interests of others but are available from the corresponding author on reasonable request.
